# An Unusual Case of Eosinophilic Chronic Rhinosinusitis With Concurrent Eosinophilic Otitis Media: Limited Responsiveness to Multiple Antibody Therapies and Subsequent Cochlear Implantation

**DOI:** 10.7759/cureus.49033

**Published:** 2023-11-18

**Authors:** Kosuke Urabe, Yumi Ohta, Soichiro Fujii, Takeshi Tsuda, Hidenori Inohara

**Affiliations:** 1 Otorhinolaryngology, Head and Neck Surgery, Graduate School of Medicine, Faculty of Medicine, Osaka University, Suita, JPN

**Keywords:** eom, ecrs, eosinophilic otitis media, eosinophilic chronic rhinosinusitis, cochlear implantation, antibody resistance

## Abstract

Eosinophilic chronic rhinosinusitis (ECRS) and eosinophilic otitis media (EOM) are debilitating inflammatory conditions that affect the paranasal sinuses and middle ear, respectively, and are characterized by eosinophilic infiltration. This study describes a rare and intricate case of a 65-year-old male patient concurrently afflicted with ECRS, EOM, and bronchial asthma. Despite the systematic administration of corticosteroids and various antibody drugs, the patient's condition remained unimproved, necessitating a cochlear implant for EOM, which is seldom an aggressive intervention. The patient had a history of symptoms dating back to 2005, with notable exacerbations and treatment resistance over the years. Multiple antibody drugs, including anti-IgE, anti-IL-5, and anti-IL-4α antibodies, failed to ameliorate the patient's condition, presenting a significant clinical challenge. Pathological examination revealed marked eosinophilic infiltration and severe fibrosis, suggesting a possible mechanism underlying the poor response to antibody therapy. Cochlear implantation significantly enhanced the patient's communicative abilities. This case highlights the limitations of the current antibody drugs in managing severely intertwined cases of ECRS, EOM, and bronchial asthma, highlighting the need for novel therapeutic strategies. This case also propounds cochlear implantation as an efficacious intervention for refractory EOM with severe sensorineural hearing impairment, extending the spectrum of treatment modalities for such challenging scenarios. This singular case contributes to the growing body of evidence regarding the management of ECRS and EOM, especially against the backdrop of treatment resistance, and can aid clinicians in identifying and navigating similar complex cases in clinical practice.

## Introduction

Eosinophilic chronic rhinosinusitis (ECRS) and eosinophilic otitis media (EOM) are inflammatory conditions of the paranasal sinus and middle ear, respectively. These conditions are characterized by the infiltration of eosinophils into the affected tissues. ECRS is a subtype of chronic rhinosinusitis with nasal polyps associated with severe eosinophilic infiltration [1-3]. The incidence of non-ECRS is decreasing in East Asia, whereas that of ECRS is increasing. Similar to ECRS, EOM is a refractory otitis media associated with type 2 inflammation [4].

The treatment strategies for both ECRS and EOM aim to control local and systemic eosinophilic inflammation. Treatment options for ECRS include endoscopic sinus surgery (ESS) and the administration of systemic glucocorticoids. In addition, antibody therapies have been used to treat recurrent ECRS with considerable improvements [5,6]. Local treatment modalities for EOM include the instillation of triamcinolone acetonide or saline-heparin solution into the tympanic cavity, whereas surgical intervention may be required to manage otorrhea in patients with EOM [7,8].

The objective of this study was to report a rare case of ECRS and EOM complicated by bronchial asthma in which systematic corticosteroid therapy and multiple antibody drugs failed to improve the patient’s condition, leading to the insertion of a cochlear implant for the EOM.

## Case presentation

A 65-year-old male with a chief complaint of nasal obstruction and nasal discharge underwent ESS at the hospital’s otorhinolaryngology department in 2005. In 2007, he experienced right-sided hearing loss and earache; therefore, he visited a local otorhinolaryngology clinic and underwent a myringotomy at the same clinic. After myringotomy, the patient was treated with steroid ear drops at another nearby clinic because of a remnant perforation and intractable otorrhea associated with granulation. However, his symptoms did not improve, and in 2013, he was referred to our department for close examination and treatment because of new findings of left-sided hearing loss and otorrhea.

**Figure 1:**
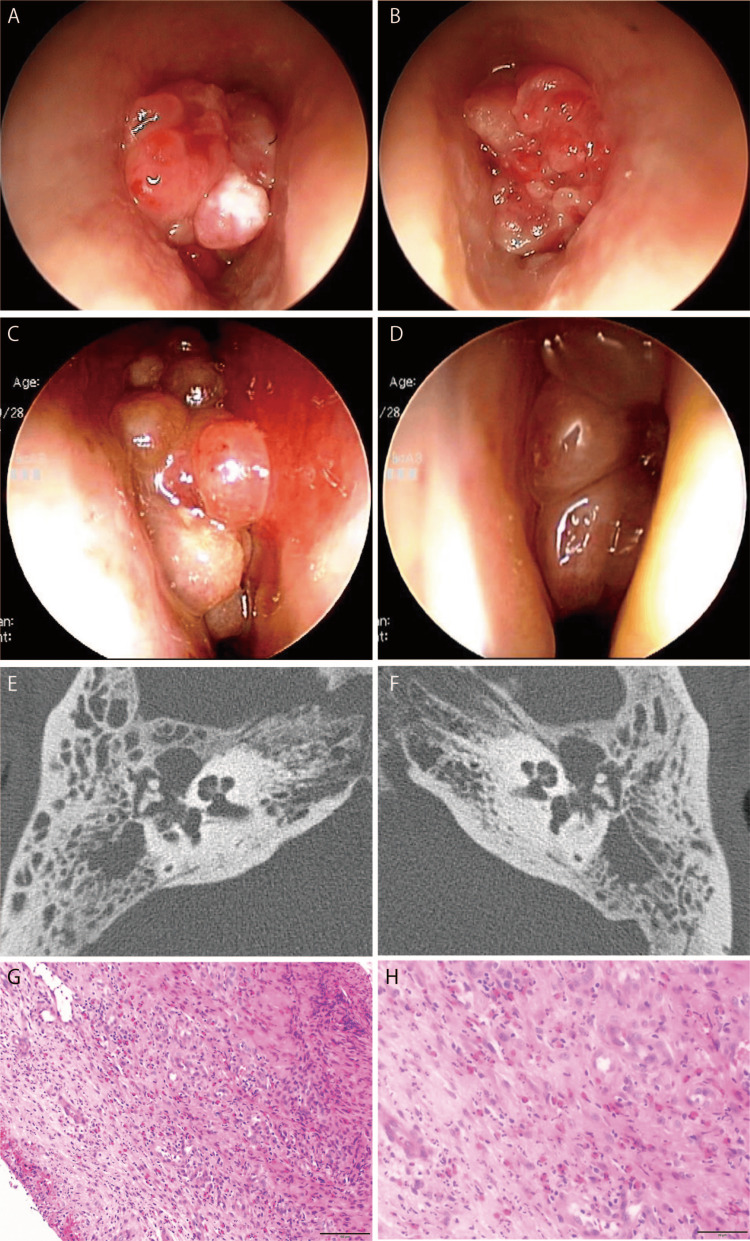
Physical examination findings at the time of the initial visit (A, B) Initial tympanic membrane findings. Granulations arising from the tympanic membrane are visible on both sides. (C, D) Nasal endoscopy findings. Nasal polyps extend from the middle nasal meatus. (E, F) Computed tomography imaging of the head shows soft shadows in the middle ear cavity. Soft shadows are visible in the middle ear cavity and throughout the paranasal sinuses, with a focus on the ethmoid sinuses on both sides. (G, H) Pathological findings of middle ear granulation and nasal polyps. Both tissues show marked eosinophil infiltration. (D)

At the initial visit, the nose was filled with nasal polyps, and granulation was detected in the bilateral tympanic membranes (Figures 1A-1D). Computed tomography (CT) imaging of the paranasal sinuses and middle ear showed soft-tissue lesions in all sinuses bilaterally and in the middle ear cavity. No apparent bone destruction was observed (Figures 1E, 1F). Biopsies of the middle ear granulation tissue revealed marked eosinophilic infiltration in histological sections, leading to the diagnosis of EOM (Figures 1G, 1H).

**Figure 2:**
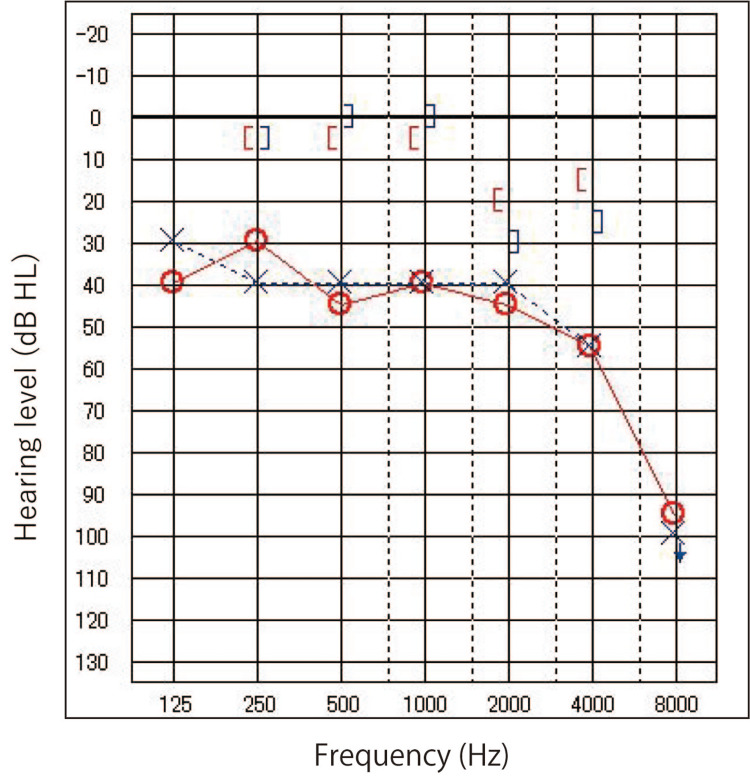
Pure tone audiometry at the time of the initial visit Mixed hearing loss on both sides

Hearing tests revealed mixed bilateral hearing loss (Figure 2).

In addition, the proportion of blood eosinophils was 17.2%, the blood eosinophil count was 1742, and the total IgE level was 121.8 IU/mL. The patient also had bronchial asthma; therefore, his primary care physician prescribed an inhaled steroid and adrenergic beta-stimulant combination drug, a leukotriene antagonist, oral theophylline, and oral steroids to manage his symptoms. After the initial diagnosis, during follow-up in our hospital, exacerbations of hearing loss were observed several times a year; therefore, intravenous corticosteroids were administered. However, in 2015, the patient's left ear lost hearing. The nasal polyps were also mildly enlarged, and biopsy results led to the diagnosis of ECRS. After discussing with a respiratory physician the effect on the granulation of the right middle ear and unstable bronchial asthma, omalizumab (an anti-IgE antibody) was initiated in the same year. The patient's condition exhibited recurrent improvements and worsening; therefore, mepolizumab (an anti-IL-5 antibody) was started in 2017 in accordance with the worsening of EOM. However, his right-sided hearing deteriorated, and he became deaf in 2020. Therefore, dupilumab (an anti-IL-4α antibody) therapy was started, which led to an exacerbation of bronchial asthma one month after the treatment initiation. Consequently, the patient was reintroduced to anti-IgE antibody therapy, which was the most stable of all the previous therapies.

**Figure 3:**
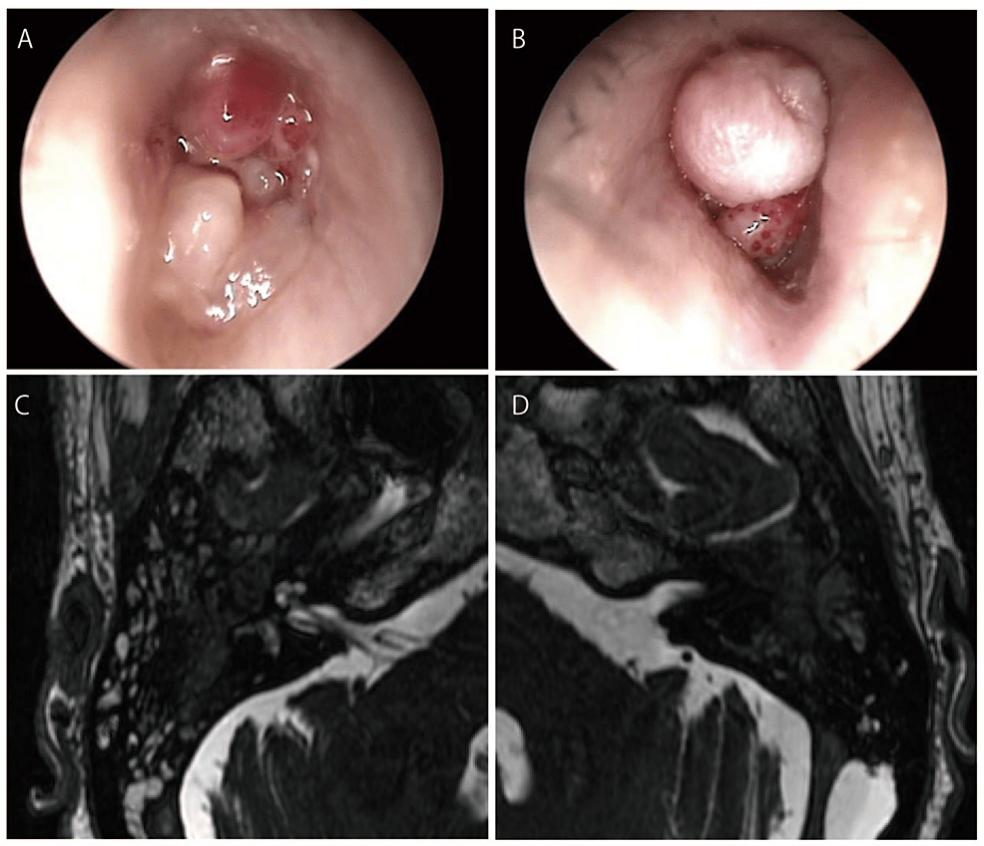
Physical examination findings before cochlear implantation (A, B) Cochlear implantation of the tympanic membrane. Increased granulation filled the ear canal compared with the initial examination. (C, D) Preoperative magnetic resonance imaging. The left cochlea is filled with soft tissue. In contrast, residual fluid is present in the right cochlea.

Thereafter, middle ear granulation continued to increase, otorrhea persisted, and magnetic resonance imaging (MRI) showed evidence of granulation filling the left cochlea (Figures 3A-3D).

**Figure 4:**
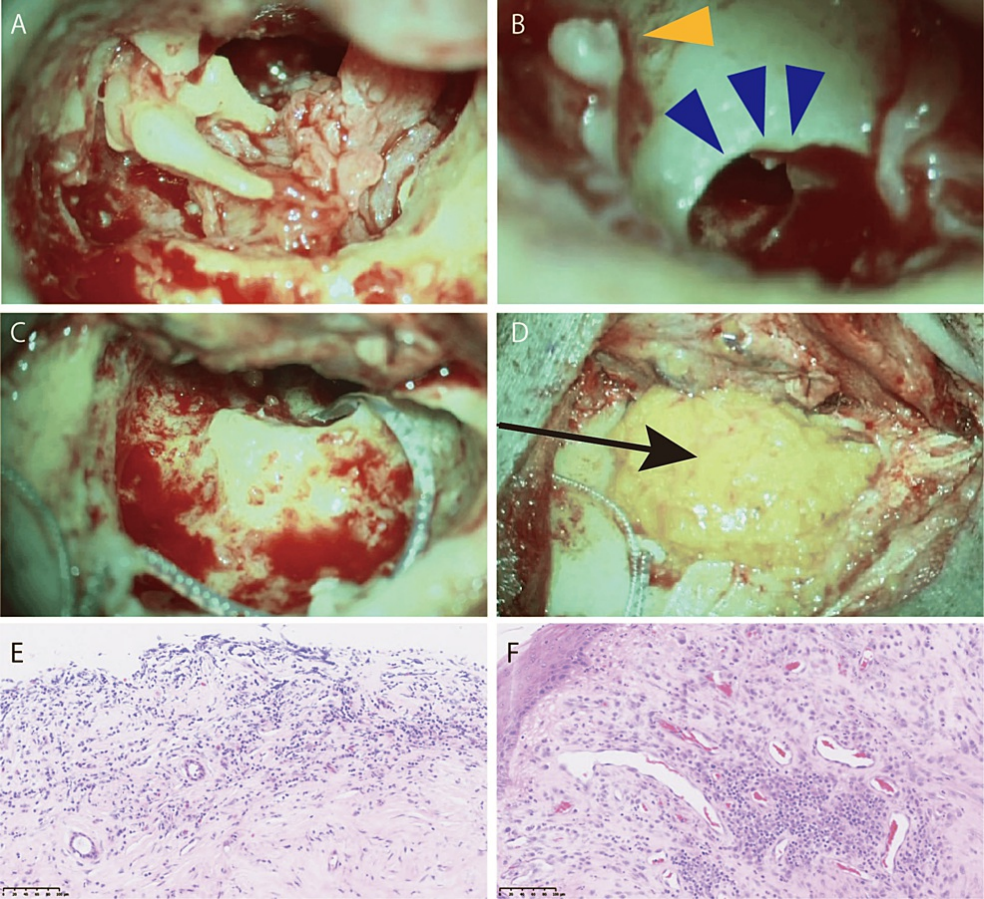
Intraoperative view of cochlear implantation (A) The middle ear cavity is filled with granules, some of which enter the cochlea. (B) Granulation was excised from the round window niche, and granulation within the cochlea was removed after shaving the bone. Stapes (yellow triangle) and enlarged area from the round window niche (blue triangle). (C) The granules were removed, and all the electrode leads of the cochlear implant were inserted without resistance. (D) The middle ear cavity is filled with fat (black arrow). (E, F) Pathological examination of the middle ear mucosa. Minimal eosinophil infiltration, whereas fibrosis is severe.

However, since we identified a liquid component in the right cochlea, the patient underwent total mucosal stripping of the right middle ear, fat filling, canalostomy, eustachian tube closure, and cochlear implantation (Nucleus® Profile™ Plus CI622) in 2021 to stop the otorrhea and reacquire vocal communication (Figures 4A-4D). The middle ear mucosa removed during surgery was infiltrated with inflammatory cells such as neutrophils, although eosinophil infiltration was not evident, and significant tissue fibrosis was observed (Figures 4E, 4F).

**Figure 5:**
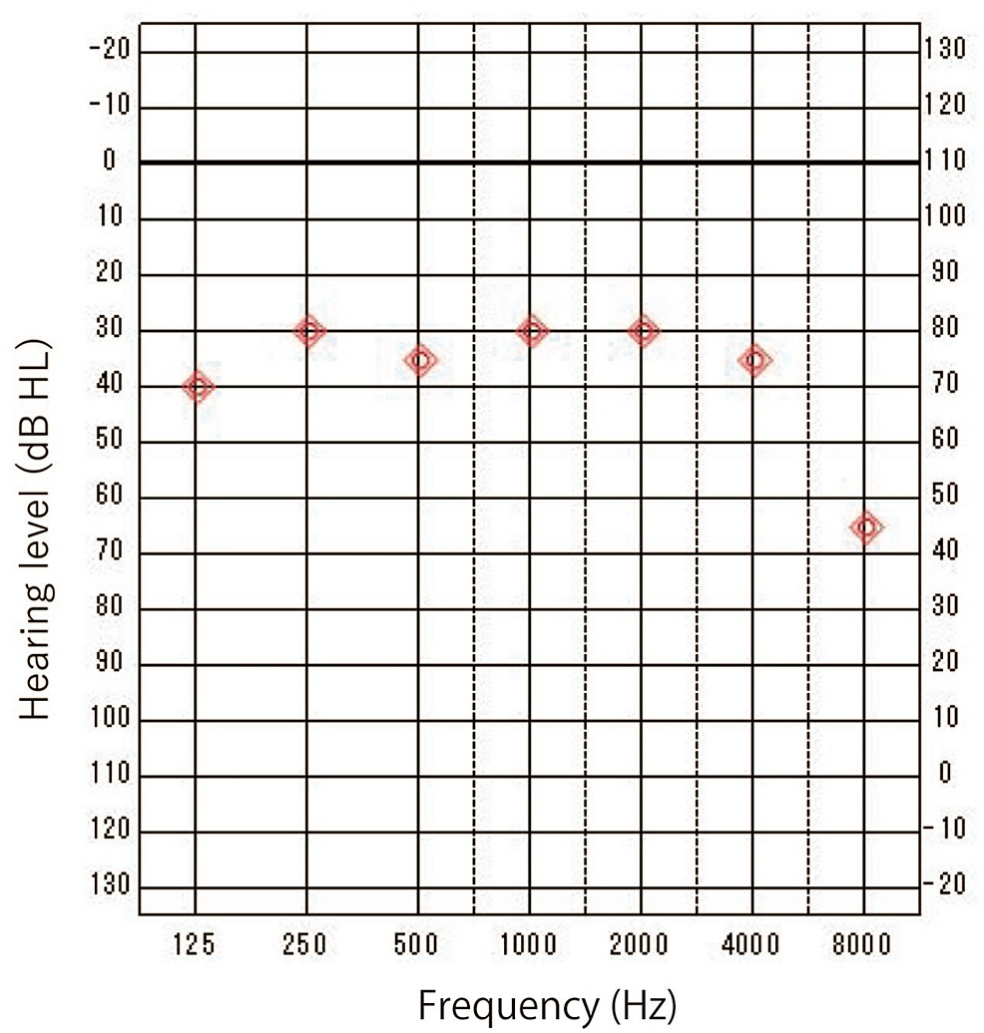
Latest auditory examination (the threshold with CI) Good hearing performance was recognized CI: cochlear implant

The patient was able to communicate well with the cochlear implant. Hearing thresholds (with cochlear implant (CI)) were 30 dB, and speech word recognition in quiet was 58.3% (auditory), and 86.7% (auditory + visual). Figure 5 shows the details of the hearing test two years after surgery.

**Figure 6:**
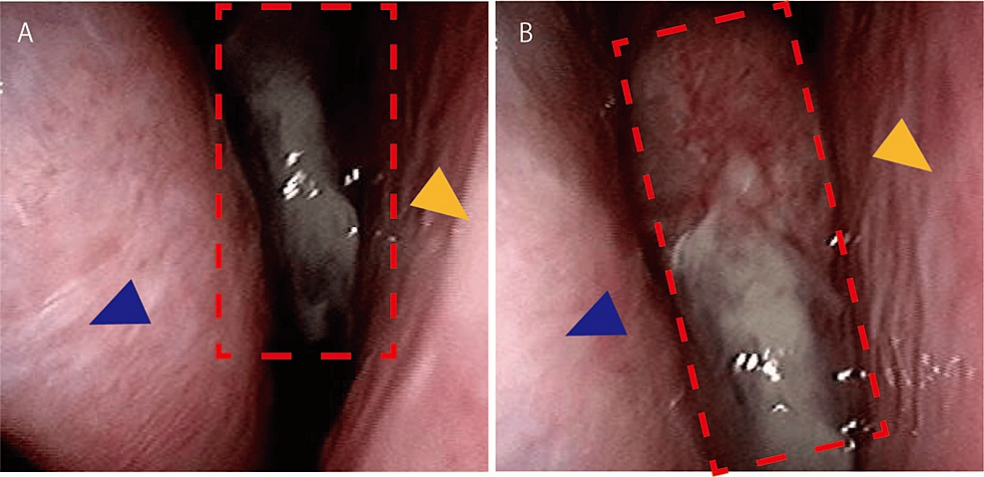
Current intranasal fiber findings Current intranasal findings. Nasal polyps (red dash square) are present in the nose; however, some spaces are also visible in the common nasal passages. Septum (yellow arrow) and inferior turbinate (blue arrow).

Regarding nasal cavity findings, the nasal polyps were not reduced during the administration of various antibody drugs (Figure 6).

As the patient's symptoms were minimal, no further surgery was performed, and the patient was followed up on an outpatient basis.

## Discussion

In this study, we present an uncommon case of ECRS and EOM complicated by bronchial asthma. Despite the administration of multiple antibody medications, the patient’s condition did not improve, necessitating the placement of a cochlear implant for EOM. This case illustrates the challenges and limitations of current treatment strategies for ECRS and EOM and highlights the need for further research and the development of novel therapies for these conditions.

A notable feature in this case is that the patient responded poorly to various antibody drugs, including anti-IgE, anti-IL-5, and anti-IL-4α antibodies. These drugs have been shown to effectively reduce eosinophilic inflammation and improve symptoms in patients with ECRS and EOM [5,6,9-11]. Previous reports have not identified factors predicting poor responses to antibody drugs for ECRS or EOM. In particular, in cases of ECRS, subgroup analyses of various clinical findings did not identify any poor responders, as all groups showed a treatment response. However, in our case, none of these drugs prevented the recurrence of hearing loss or otorrhea or reduced the nasal polyps or granulation tissue in the ear. In type 2 inflammatory diseases, such as EOM, fibrosis is thought to be enhanced by tissue damage through eosinophil etosis and by the binding of periostin induced by inflammatory cytokines to other extracellular matrices. Interestingly, pathological specimens from cochlear implantation showed few eosinophils and severe fibrosis in this case. To the best of our knowledge, there have been no reports of cases of EOM that developed fibrosis with inflammation instead of eosinophilic infiltration during the disease course. Although various antibody drugs contribute to the elimination of eosinophils and suppression of inflammatory cytokines, the mechanism of action of these drugs suggests that they have little effect on fibrosis once it has been established. Based on these considerations, severe fibrosis may be involved in the pathogenesis, causing poor antibody drug response; however, further investigation will be needed.

Another novel finding of this study is that the patient underwent cochlear implantation for EOM, a rare and aggressive treatment option for this condition. Cochlear implantation is usually reserved for patients with severe sensorineural hearing loss caused by inner ear damage or nerve degeneration. In the present case, the patient had mixed hearing loss due to conductive and sensorineural components. The conductive component was caused by granulation tissue filling the middle ear cavity, whereas the sensorineural component was caused by eosinophilic infiltration of the inner ear. The patient eventually became bilaterally deaf after recurrent improvement and worsening of mixed hearing loss, a process often observed in patients with EOM. The cochlear implantation was performed to directly stimulate the auditory nerve, and the patient was able to communicate well with the cochlear implant. However, the long-term results of cochlear implants are very important, and we plan to follow up with these patients for a longer period of time.

This case demonstrates that cochlear implantation can be a viable option for patients with refractory EOM with severe sensorineural hearing impairment, leading to a poor quality of life. Recently, a new antibody, tezepelumab (an anti-thymic stromal lymphopoietin antibody), was approved for the treatment of asthma [12]. However, given the mechanism of action of tezepelumab, it is unlikely to have a significant advantage over existing antibody drugs in patients with severe fibrosis. Surgical procedures such as ESS and cochlear implantation are expected to continue to play an important role in improving patients' quality of life.

One limitation of this study was that it was based on a single case report, which may not represent the general population of patients with ECRS and EOM. Therefore, caution should be exercised when generalizing the findings of this study to other settings. Moreover, this study did not perform any molecular or immunological analyses of the patient’s tissues or blood samples, which could have provided more insight into the pathogenesis and treatment response of these conditions.

## Conclusions

We report a rare case of ECRS and EOM complicated by bronchial asthma in which multiple antibody drugs failed to improve the patient’s condition, leading to the insertion of a cochlear implant for EOM. Antibody drugs are highly effective in the treatment of refractory type 2 inflammatory diseases, and drug rotation is possible even if a particular formulation is ineffective. However, some patients respond poorly to multi-antibody drugs. This case adds to the existing literature on these conditions and may help clinicians recognize and manage similar cases in the future. Furthermore, this case illustrates the challenges and limitations of current treatment strategies for ECRS and EOM while highlighting the need for further research and development of novel therapies for these conditions.
